# Cell cycle activation contributes to isoflurane‐induced neurotoxicity in the developing brain and the protective effect of CR8

**DOI:** 10.1111/cns.13090

**Published:** 2019-01-24

**Authors:** Bao‐Yi Huang, Hong‐Bing Huang, Zhi‐Jing Zhang, Zhi‐Gang Liu, Jun Luo, Min Liu, Tao Luo

**Affiliations:** ^1^ Department of Anesthesiology Peking University Shenzhen Hospital Shenzhen China; ^2^ Shantou University Medical College Shantou Guangdong P.R. China; ^3^ Sun Yat‐sen University Cancer Center State Key Laboratory of Oncology in South China Collaborative Innovation Center for Cancer Medicine Guangzhou China; ^4^ Department of Anesthesiology Renmin Hospital of Wuhan University Wuhan China; ^5^ Department of Pathology Zhongnan Hospital of Wuhan University Wuhan China; ^6^ Health and Family Planning Capacity Building and Continuing Education Center of Shenzhen Municipality Shenzhen China

**Keywords:** apoptosis, CR8, cyclin B1, isoflurane

## Abstract

**Aims:**

It is well established that exposure of common anesthetic isoflurane in early life can induce neuronal apoptosis and long‐lasting cognitive deficit, but the underlying mechanisms were not well understood. The cell cycle protein Cyclin B1 plays an important role in the survival of postmitotic neurons. In the present study, we investigated whether cyclin B1‐mediated cell cycle activation pathway is a contributing factor in developmental isoflurane neurotoxicity.

**Methods:**

Postnatal day 7 mice were exposed to 1.2% isoflurane for 6 hours. CR8 (a selective inhibitor of cyclin‐dependent kinases) was applied before isoflurane treatment. Brain samples were collected 6 hours after discontinuation of isoflurane, for determination of neurodegenerative biomarkers and cell cycle biomarkers.

**Results:**

We found that isoflurane exposure leads to upregulated expression of cell cycle‐related biomarkers Cyclin B1, Phospho‐CDK1(Thr‐161), Phospho‐n‐myc and downregulated Phospho‐CDK1 (Tyr‐15). In addition, isoflurane induced increase in Bcl‐xL phosphorylation, cytochrome c release, and caspase‐3 activation that resulted in neuronal cell death. Systemic administration of CR8 attenuated isoflurane‐induced cell cycle activation and neurodegeneration.

**Conclusion:**

These findings suggest the role of cell cycle activation to be a pathophysiological mechanism for isoflurane‐induced apoptotic cell death and that treatment with cell cycle inhibitors may provide a possible therapeutic target for prevention of developmental anesthetic neurotoxicity.

## 1 INTRODUCTION

Millions of children undergo surgical operations or diagnostic procedures with general anesthesia across the world each year.^1^ Over the past decade, an increasing number of reports from preclinical investigations have found that exposure to common anesthetic agents (eg, isoflurane and sevoflurane) during vulnerable periods of brain development results in widespread cellular toxicity including apoptosis and neurodegeneration, which in turn leads to neurocognitive impairment and decreased neuronal density in adults.^1^, ^2^ Among the clinically used general anesthetic drugs, isoflurane is more likely to cause widespread neurodegeneration in the newborn rodents, pigs, or non‐human primates.^3^, ^4^, ^5^, ^6^ But the specific molecular mechanisms and pathways of isoflurane‐induced neurotoxicity in the developing brain still remain to be elucidated.

In proliferating cells, the cell cycle is governed by a complex network of signaling pathways, and progression through different phases requires sequential activation of a large group of cell cycle regulatory molecules.^7^ Neurons, usually regarded as postmitotic cells, are considered to arrest in the G0 phase and unable to proliferate.^8^ Recent studies have suggested a relationship between aberrant cell cycle progression and neuronal apoptosis in the developing nervous system, as well as neural degeneration in distinct models of adult brain following stroke and trauma, epilepsy, Alzheimer’s disease, Parkinson’s disease, and amyotrophic lateral sclerosis.^9^, ^10^, ^11^, ^12^, ^13^, ^14^, ^15^, ^16^, ^17^ Cell cycle inhibition offers neuroprotection both in vivo and in vitro.^18^, ^19^, ^20^, ^21^, ^22^

Cyclin B1 is a cell cycle protein involved in the G2/M transition and plays a vital role in the survival of postmitotic neurons.^23^ In healthy neurons, cyclin B1 degradation takes place in the proteasome after ubiquitylation via the complex/cyclosome (APC/C)‐cadherin 1 (Cdh1).^24^ In the pathophysiological situation of Alzheimer’s disease,^25^ experimental brain ischemia,^26^ traumatic brain injury,^19^ however, the affected brain regions aberrantly express cyclin B1. Accumulation of cyclin B1 leads to activation of cyclin B1‐Cdk1(cyclin‐dependent kinase 1) complex, which is responsible for the increased phosphorylation of Bcl‐xl and Bcl‐2, and contributes to subsequent activation of the intrinsic cell death pathway^23^, ^27^ Although previous studies have identified cyclin B1/ Bcl‐X_L_ as a key pathway in neuronal apoptosis, the potential role of this signal transduction pathway in the regulation of neuronal cell death following isoflurane anesthesia in developing brain has not been addressed.

We hypothesized that isoflurane induces cyclin B1‐mediated cell cycle activation, contributing to general anesthesia related neuronal cell death in the developing brain, and that inhibiting this pathway by a novel potent selective CDK inhibitor, CR8, reduces isoflurane‐induced neuronal cell death. In the present study, we tested these two hypotheses, using a well‐established experimental model of isoflurane‐induced neurotoxicity in neonatal mice.

## 2 MATERIALS AND METHODS

### 2.1 Animals and experimental procedure

All the animal protocols were approved by the Institutional Animal Care and Use Committees. C57BL/6 mice (n = 45, obtained from Model Animal Research Center of Nanjing University, Nanjing) were housed with food and water available ad libitum and maintained under defined protocol with a 12 hours light‐dark cycle.

In the isoflurane exposure study, mice at postnatal day 7 (P7) were placed inside a clear plexiglass chamber, resting on a heating pad to maintain their body temperature at about 37°C, and received 1.2% isoflurane in 30% oxygen/air at 5 L/min for 6 hours continuously. Mice in the control group were exposed to 30% oxygen/air only for 6 hours at room temperature. In the CR8 treatment study, mice were intraperitoneally administered 1 mg/kg of CR8 or an equal amount of vehicle 30 minutes before exposure to isoflurane. The dose of CR8 was selected based on previous in vivo study showing brain neuroprotection of CR8 after spinal cord injury.^28^ The concentration of isoflurane, oxygen, and carbon dioxide in the chamber was continuously monitored using an anesthetic agent analyzer. All animals were closely inspected for respiratory effort and skin color. Isoflurane treatment of up to 1.5% for 6 hours had been shown not to cause hypoxia in our pilot study as well as previous report.^5^

### 2.2 Tissue collection and western blot

At 6 hours after discontinuation of isoflurane, mouse pups were euthanized; the brains were immediately removed; and the cerebral cortices (an area particularly prone to neuronal apoptosis)^6^ were harvested, frozen in liquid nitrogen, and stored at −80°C. The samples were then lysed in RIPA buffer, and proteins were separated by SDS‐PAGE and electrophoretically transferred to a PVDF membrane. The immunoblots were probed with an anti‐cyclin B1 (Cell Signaling Technology, Danvers, MA, USA Catalog #4138). Phospho‐cdc2 (Thr161) (Cell Signaling Technology, Catalog # 9114), Phospho‐cdc2 (Tyr15) (Cell Signaling Technology, Catalog # 9111), phospho‐n‐myc (Bethyl Laboratories, Montgomery, TX, USA Catalog # A300‐206A), Bcl‐xL (Cell Signaling Technology, Catalog # 2762), Bax (Cell Signaling Technology, Catalog # 2772), Phospho‐Bcl‐xL (Ser62) (Thermo Fisher Scientific, Waltham, MA, USA Catalog # 44‐428G), Cytochrome c (Cell Signaling Technology, Catalog # 4272), and Cleaved Caspase‐3 (Asp175) (Cell Signaling Technology, Catalog # 9661). *β*‐Actin (Cell Signaling Technology, Catalog # 4967) was used as an endogenous control. Membranes were scanned and captured using an Odyssey detection system (LI‐COR Biosciences, Lincoln, NE, USA). Protein band intensities were quantified by densitometric analysis with ImageJ software and normalized according to endogenous control for each sample.

### 2.3 Tissue processing and immunohistochemistry

At 6 hours after discontinuation of isoflurane, the mice were euthanized with deep anesthesia and perfused transcardially with saline followed by 4% paraformaldehyde in Tris‐buffered saline. The brains were removed and postfixed in 4% PFA overnight and transferred to 30% sucrose in PBS for cryoprotection. Frozen brains were cut on a cryostat microtome at −18°C into 20‐μm coronal sections. Fluoro‐Jade B (Merck Millipore, Billerica, MA, USA Catalog # AG310) staining was performed to detect degenerating neurons, according to the manufacturer’s protocol. For immunocytochemistry, sections were processed and stained, using one or more antibodies recognizing Cyclin B1 (Cell Signaling Technology, Catalog # 4138), and the neuronal marker: NeuN (Cell Signaling Technology, Catalog #24307). Images were acquired with an Olympus BX53 microscope equipped with an Olympus DP47 camera at ×200 magnification. The number of caspase‐3, cyclin B1, and Fluoro‐Jade B positive cells per mm^2^ in the cerebral cortex, as well as hippocampus of each brain slice were counted by a blinded examiner. A total of four sections were counted per animal.

### 2.4 Statistical analyses

All data are presented as the mean ± SEM. Statistical analyses were performed with the GraphPad Prism 5 software using Student’s *t*‐test for unpaired data or one‐way ANOVA with the Newman‐Keuls post hoc test whenever appropriate. *P *< 0.05 were regarded statistically significant.

## 3 RESULTS

### 3.1 Postnatal exposure to isoflurane induced brain caspase‐3 activation

**Figure 1:**
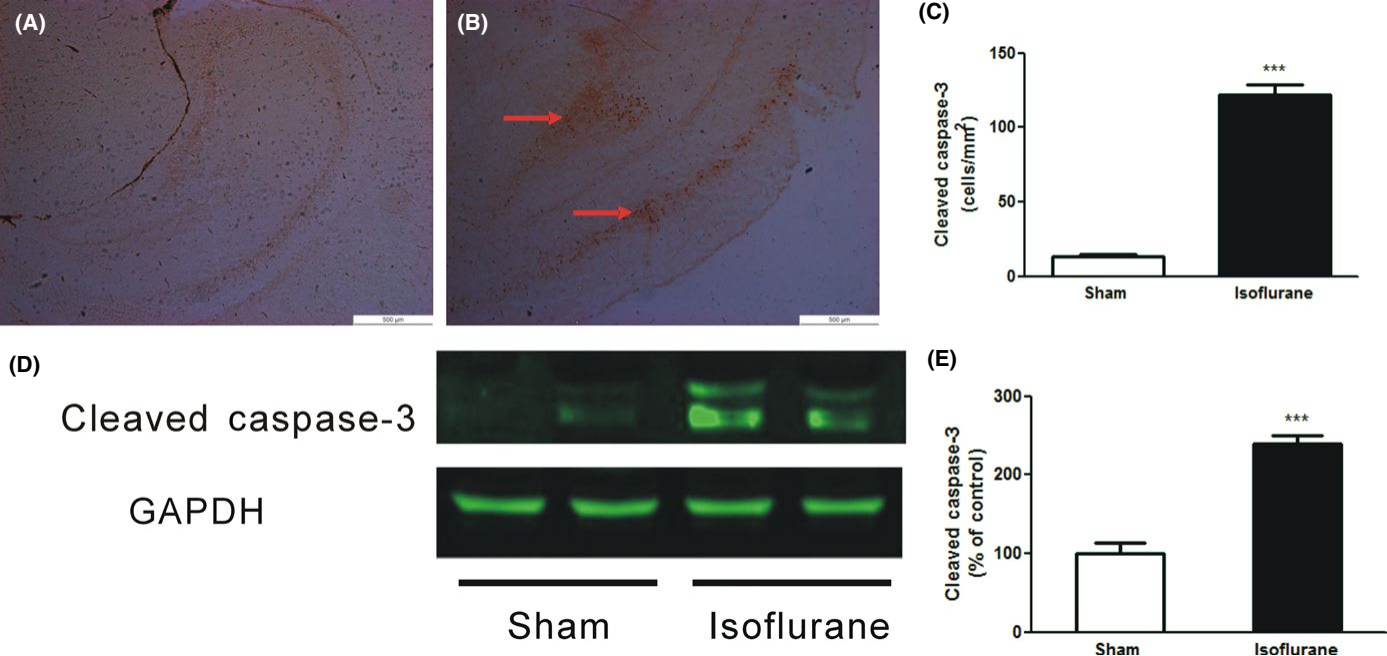
Isoflurane induces caspase‐3 activation in neonatal mice. (A‐B) Representative immunohistochemistry studies show the expression of cleaved caspase‐3 (*brown*) positive cells in the brain tissues (scale bar = 500 μm) of postnatal day 7 (P7) mouse pups exposed to either 30% oxygen/air (A) or 1.2% isoflurane (B) for 6 hours. (C) Quantitation of cleaved caspase‐3 immunopositive cells in cerebral cortex and hippocampus demonstrates greater number of cleaved caspase‐3 positive cells in mouse pups receiving isoflurane. (D) Representative immunoblots for cleaved caspase‐3 and the loading control (GAPDH) in animals treated with either 30% oxygen/air or 1.2% isoflurane for 6 hours. (E) Quantitative analysis of western blots shows that cleaved caspase‐3 levels were significantly increased after exposure to isoflurane in the P7 mouse brain. Data presented are mean ± SEM compared with sham (n = 4‐6, ***P < 0.001)

Caspase‐3 activation plays a central role in the process of apoptosis, and the cleavage of caspase‐3 is a well‐established biomarker for cell death by apoptosis. We therefore set out to determine the expression of cleaved caspase‐3 positive cells in the brain tissues of postnatal day 7(P7) mouse pups receiving either air/oxygen or 1.2% isoflurane for 6 hours. As shown in Figure 1A‐C, the number of cleaved caspase‐3 cell was 13 ± 2/mm^2^ when mouse pups were exposed to air/oxygen; however, administration of 1.2% isoflurane for 6 hours resulted in a significant increase of cleaved caspase‐3 expression (122 ± 7/mm^2^; *P *<* *0.0001 vs sham). Similarly, immunoblotting revealed that isoflurane exposure led to a 239% (*P *<* *0.0001) increase in cleaved caspase‐3 protein levels at 6 hours after anesthesia (Figure 1D, E). Thus, the changes of cleaved caspase‐3 expression with both immunohistochemistry and immunoblotting assay validated that isoflurane induced significant apoptosis in the neonatal mouse brain.

### 3.2 Isoflurane induced cell cycle‐related protein activation

**Figure 2:**
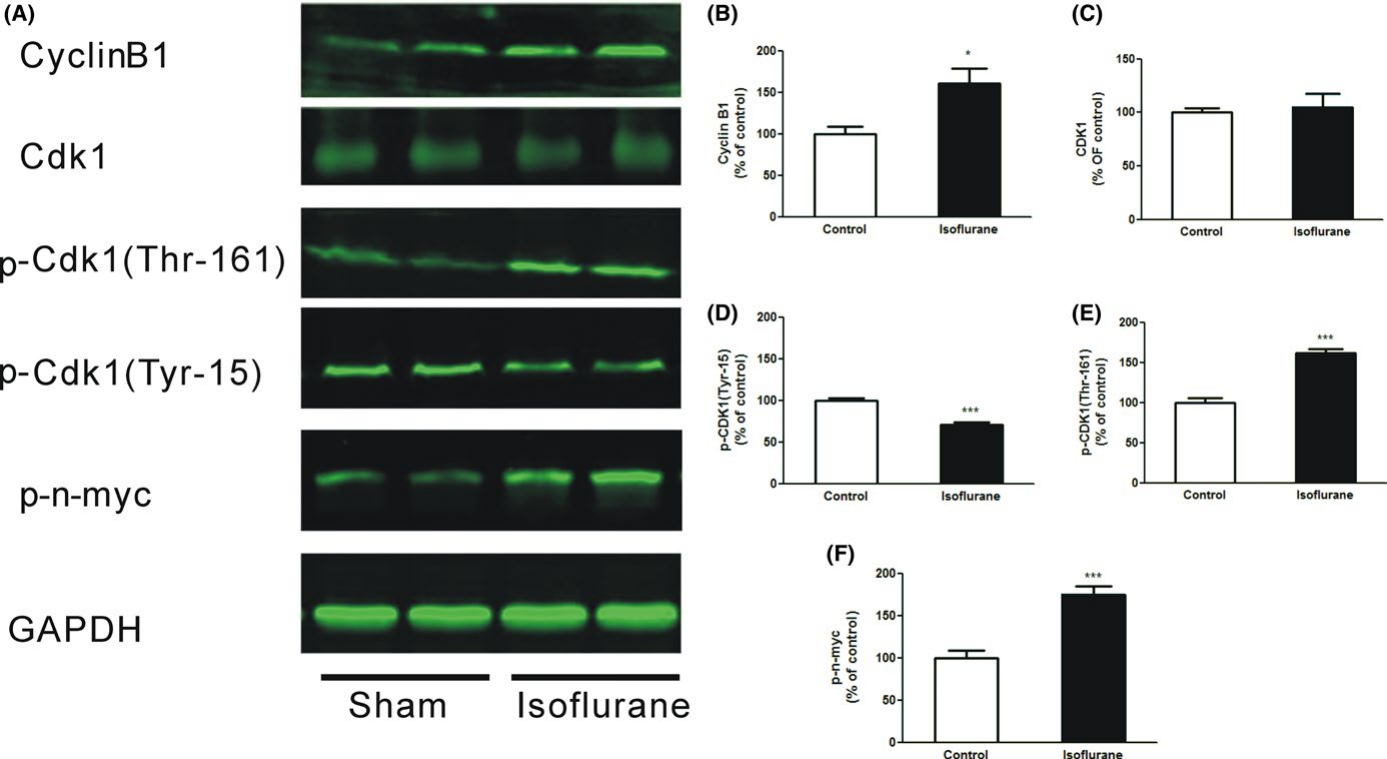
Effects of isoflurane on cell cycle‐related proteins expression. (A) Representative immunoblotting of cell cycle‐related proteins in animals exposed to 1.2% isoflurane for 6 hours. (B‐F) Quantitative analysis of western blots shows the expression of cell cycle‐related proteins cyclin B1(B), CDK1 (C), phospho‐Cdk1(Tyr‐15) (D), phospho‐Cdk1(Thr‐161) (E), and phospho‐n‐myc (F) in the mouse brain after exposure to isoflurane or 30% oxygen/air, normalized to GAPDH levels. Data presented are mean ± SEM compared with sham (n = 4‐6, *P <* *0.05, ***P <* *0.001)

Continuous degradation of cyclin B1 and inactivation of CDK1/cyclin B1 complex are essential for the survival of postmitotic neurons. To examine whether the cyclin B1‐mediated pathway was involved in neurotoxicity by isoflurane, the protein level of Cdk1, phospho‐CDK1(Thr‐161), phospho‐CDK1(Tyr‐15), and phospho‐n‐myc was evaluated by Western blot analysis. There was significant upregulation of cyclin B1 (Figure 2A, B; *P *=* *0.01 vs sham) at 6 hours after isoflurane exposure. Cyclin B binds to CDK1 to form the cyclin B/CDK1 complex, which is the key event that initiates mitotic entry. While total CDK1 levels remained relatively stable (Figure 2A, C; *P *>* *0.05 vs sham), the levels of phosphorylated CDK1 at Tyr‐15 appeared to be downregulated (Figure 2A, D; *P *<* *0.0001 vs sham), and the levels of phosphorylated Cdk1 at Thr‐161 were upregulated in isoflurane‐treated animals compared to animals exposed to air alone (Figure 2A, E; *P *<* *0.0001 vs sham). At the same time, the level of CDK1 substrate n‐myc phosphorylated at Ser (54) was upregulated following isoflurane exposure (Figure 2A, F; *P* =0.0001 vs sham), demonstrating increased CDK1 activation. Taken together, these results indicated that isoflurane exposure contributed to cell cycle‐related genes expression, which was one of the mechanisms for inhalational anesthetics‐mediated developmental cell apoptosis.

### 3.3 CR8 Inhibits cell cycle and caspase‐3 activation in the cortical tissue after isoflurane exposure

**Figure 3:**
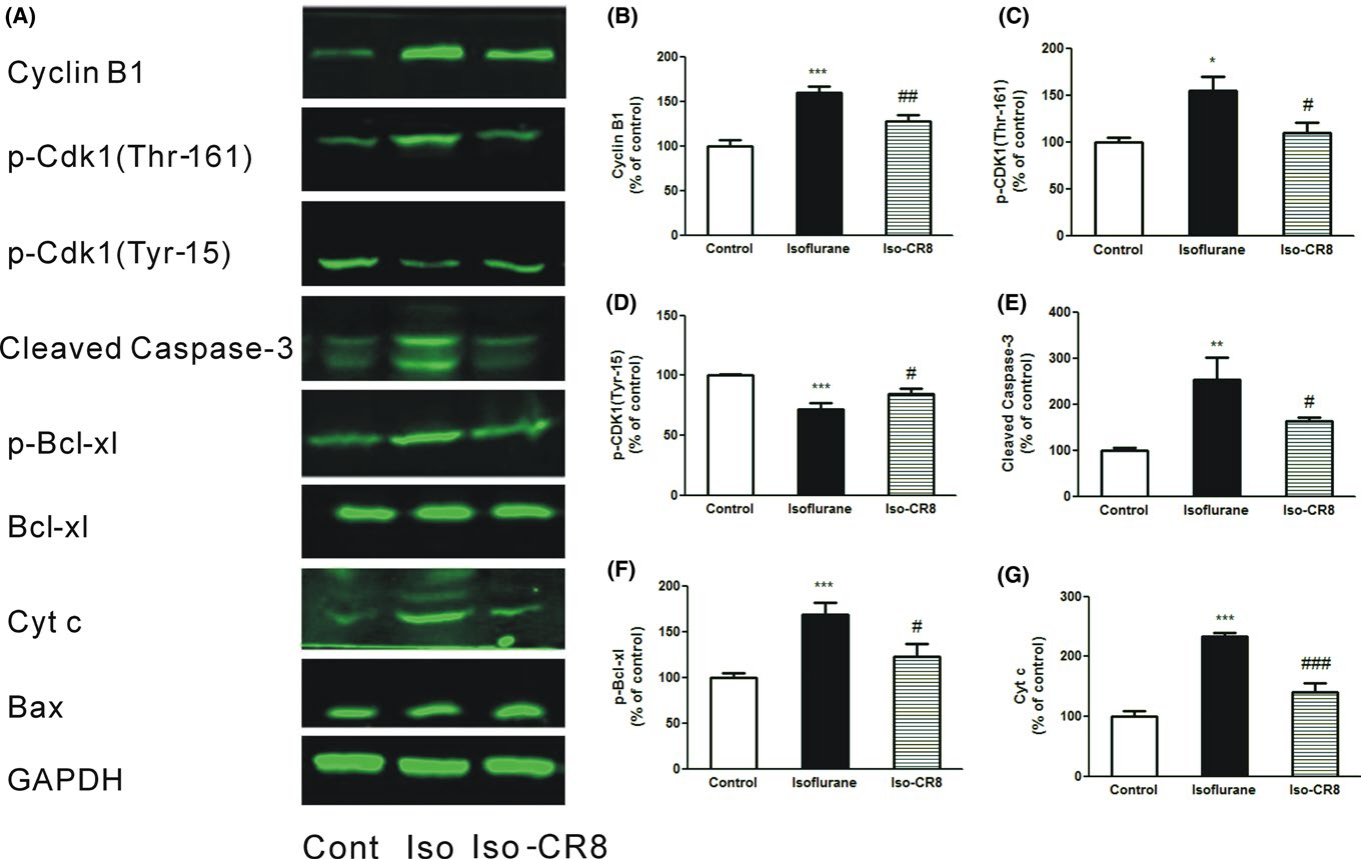
Systemic administration of CR8 inhibits isoflurane‐induced neuronal cell cycle activation and neuronal apoptosis. (A) Representative immunoblotting of cell cycle and apoptotic proteins in animals exposed to 1.2% isoflurane with or without CR8. (B‐G) Quantitative analysis of western blots shows the effect of CR8 on isoflurane anesthesia induced cyclin B1(B), phospho‐Cdk1(Thr‐161) (C), phospho‐Cdk1(Tyr‐15)(D), cleaved caspase‐3(E), phospho‐Bcl‐x(F), and cytochrome c (G) levels in the mouse brain, normalized to GAPDH levels. Data presented are mean ± SEM (n = 4‐6, *P < 0.05, **P <* *0.01, ***P < 0.001 compared with sham; #*P *<* *0.05, ##*P* < 0.01, ###*P *<* *0.001 compared with Isoflurane)

In considering that isoflurane may induce apoptosis through regulating the cell cycle‐related protein activation, we next investigated whether the isoflurane‐induced neurotoxicity can be reversed by pharmacological cell cycle inhibitor. CR8 is a selective CDK inhibitor that has been shown to attenuate neuronal cell cycle activation and neurodegeneration in a mouse isolated thoracic spinal cord contusion model.^28^ We therefore set out to determine the above activated cell cycle markers in isoflurane‐treated mouse pups with or without CR8 pretreatment. Notably, the elevated expression of Cyclin B1 (Figure 3A, B; *P *<* *0.01 vs vehicle) and p‐Cdk1(Thr‐161) (Figure 3A, C; *P *<* *0.05 vs vehicle) induced by isoflurane exposure was profoundly attenuated by systemic CR8 pretreatment, while the decreased level of p‐CDK1(Tyr‐15) (Figure 3A, D; *P *<* *0.05 vs vehicle) was rescued by CR8 pretreatment. CR8 treatment also profoundly inhibited the level of the cleaved caspase‐3 expression when compared with vehicle‐treated animals (Figure 3A, E; *P *<* *0.05 vs vehicle), indicating attenuation of cell apoptosis.

Cyclin B1/Cdk1 is capable of phosphorylating the B‐cell lymphoma extra‐large (Bcl‐xL) and induces antiapoptotic response.^23^ We further assessed the effect of CR8 on apoptotic‐related protein expression. As shown in Figure 3, Bcl‐xL phosphorylation appeared to increase following isoflurane exposure (Figure 3A, F; *P *<* *0.001 vs sham); CR8 administration significantly ameliorated these changes (Figure 3A, F; *P *<* *0.05 vs vehicle). However, the expression of Bcl‐xL and bax was not influenced by either isoflurane or CR8. Next, we studied the effects of isoflurane on cytochrome C level, a downstream factor of Bcl‐xL in brain tissues.^29^ Isoflurane exposure (Figure 3A, G; *P *<* *0.001 vs sham) induced a prominent increase of cytochrome c release as compared to the control condition, which can be attenuated by CR8 administration (Figure 3A, G *P *<* *0.001 vs vehicle).

### 3.4 Isoflurane‐induced Cyclin B1 immunostaining positive cells are mainly neurons

**Figure 4:**
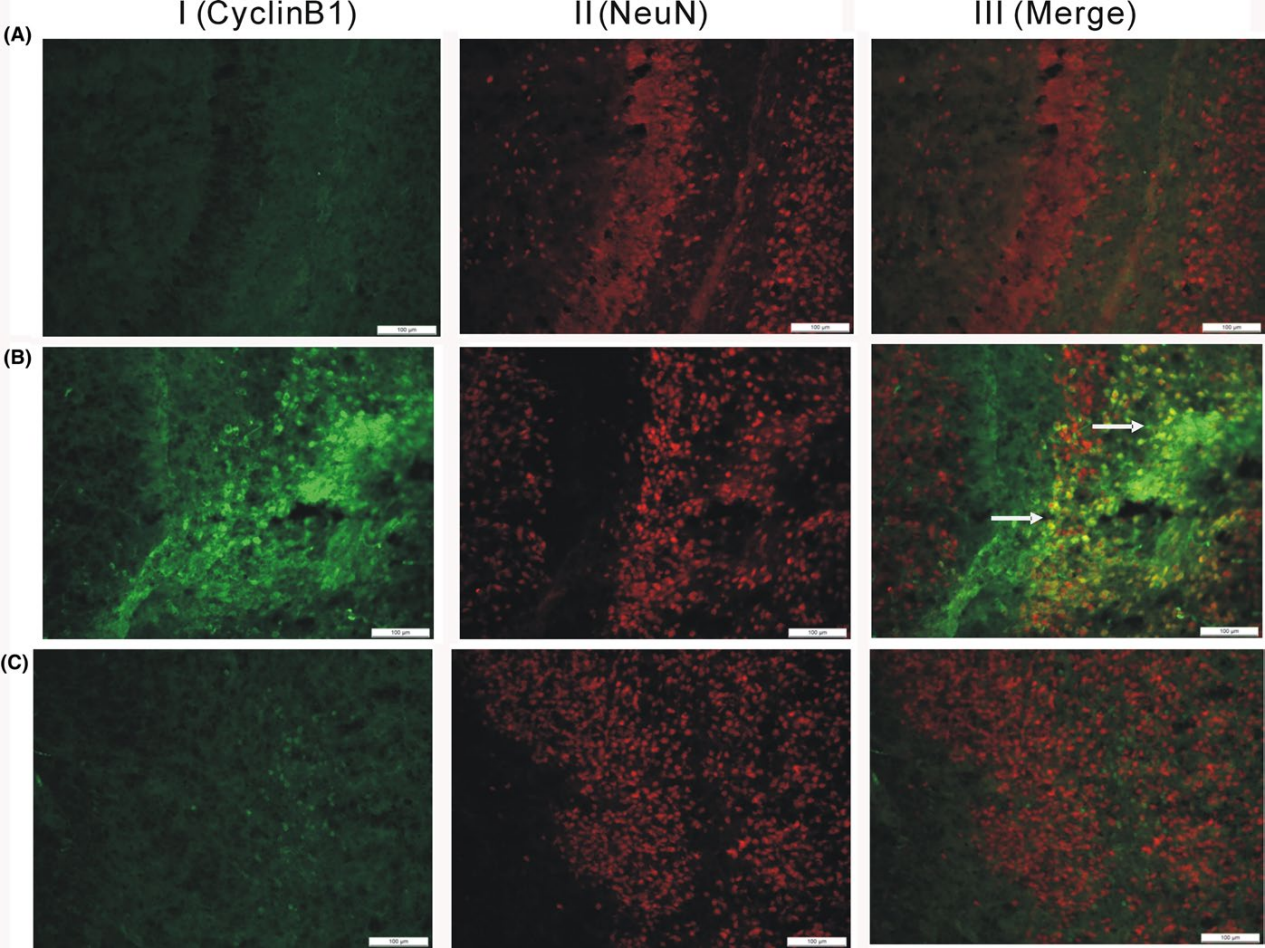
Isoflurane‐induced cyclin B1 immunostaining positive cells are confined to neurons. Representative immunostaining for cyclin B1 (green), neuronal marker NeuN (red), Merged cyclin B1 and NeuN (yellow) in the brain tissues of postnatal day 7 mouse pups exposed to either 30% oxygen/air (A), 1.2% isoflurane for 6 hours (B), and isoflurane with CR8 treatment (C). The white arrow indicates examples of cyclin B1+/NeuN+/ co‐labeled cells (yellow). (Scale bar = 500 μm)

Since cell cycle proteins are present in each cell type, and neurons are terminally differentiated, we then investigated if neurons were mainly involved in isoflurane‐induced cell cycle activation using immunofluorescence analysis. As can be seen in Figure 4,column I is the immunofluorescence imaging of cyclin B1 (green), column II is the immunofluorescence imaging of NeuN (red), and column III is the merged image. The imaging showed numerous NeuN positive cells in both cortex and hippocampus of sham‐animals, but few were co‐labeled with cyclin B1. However, following isoflurane exposure, cyclin B1 expression was also frequently detected in NeuN‐labeled (NeuN+) cells. Quantitation of cyclin B1 positive cells demonstrated an average of 187 ± 15 cells/mm^2^. Administration of CR8 reduced the number of cyclin B1+ cells to 82 ± 12 cells/mm^2^ (Figure 4, *P* < 0.001 vs vehicle). Few NeuN‐labeled cells were detected in tissue double labeled with cyclin B1 following CR8 treatment. Taken together, these results suggest that isoflurane‐induced cell cycle activation is mainly confined to neuronal cell.

### 3.5 CR8 inhibits isoflurane‐induced degeneration of neurons in the brain

**Figure 5:**
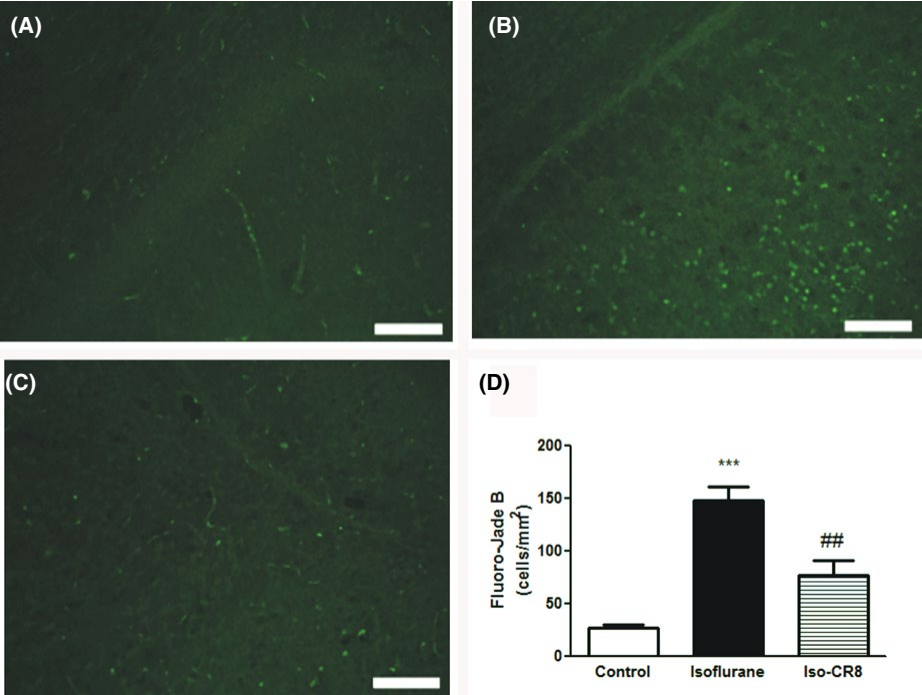
CR8 inhibits isoflurane‐induced degeneration of neurons in the postnatal mouse brain. (A‐C) Immunofluorescence photomicrographs of Fluoro‐Jade B (green) positive cells in mouse brain exposed to 1.2% isoflurane with or without CR8 (scale bar = 100 μm). (D) Quantitative analysis of neuronal degeneration in the brain as a number of neurons positively stained with Fluoro‐Jade B (degenerating neurons) in sham control, isoflurane, and isoflurane+CR8 groups. Data presented are mean ± SEM (n = 4, ***P <* *0.001 compared with sham; ##*P *<* *0.01 compared with Isoflurane)

To examine the potential protective effect of CR8 on isoflurane neurotoxicity at histological level, Fluoro‐Jade B stainings of the mice brain at 6 hours after discontinuation of isoflurane were performed. Representative immunofluorescence images were shown in Figure 5. Fluoro‐Jade B staining was significantly increased in isoflurane group, suggesting a greater number of degenerating neurons (Fluoro‐Jade B positive) compared with littermates without anesthetic exposure (Figure 5A, B, D). In contrast, animals that treated with CR8 showed less intense staining of Fluoro‐Jade B around the cortical regions (Figure 5C, D), indicating fewer degenerating neurons. (77 ± 14 vs 148 ± 13 counts/mm^2^ for CR8− and vehicle‐treated animals, respectively; *P *<* *0.01 vs vehicle).

## 4 DISCUSSION

In the current study, we observed that exposure to a commonly used general anesthetic isoflurane for 6 hours caused substantial neuronal apoptosis in the postnatal day 7 mouse brain, which involves aberrant expression of cell cycle‐related proteins. This pathway includes activation of cyclin B1 and its downstream effectors, cdk‐1, bcl‐xl, cytochrome c, and ultimately leading to caspase‐3 activation and neuronal degeneration. Further, inhibition of cell cycle activation by CR8 provides neuroprotection against isoflurane. These results demonstrate that aberrant expression of cell cycle is involved in isoflurane‐induced neurotoxicity and plays an important role in the subsequent process of neuronal apoptosis. Inhibition of this pathway offers a potential therapeutic target for isoflurane neurotoxicity.

Isoflurane exposure increased expression of cyclin B1 followed by the activation of CDK1. In addition, upregulated phospho‐n‐myc levels, indicating specific CDK1 activation, were observed at 6 hours after isoflurane exposure. Most of the cyclin B1 protein appeared to be co‐localized within NeuN+ cells (neurons). It has been well known that cyclin B1‐Cdk1 is essential for mitosis, and activation of this complex confers an important role in cell growth and proliferation. However, aberrant activation of cyclin B1‐Cdk1 plays an opposite role and triggers apoptotic signaling pathway during mitotic arrest.^30^ Transient overexpression of nondegradable cyclin B1 induces apoptotic death in the KB‐3 human carcinoma cell line and mouse embryonic fibroblasts.^31^ Notably, cyclin B1 has been found to accumulate in degenerating neurons of experimental cerebral ischemia and traumatic brain injury, as well as in patients’ brain with stroke and Alzheimer’s disease.^19^, ^25^, ^26^ In postmitotic neurons, excitotoxic stimulation triggers neuronal apoptotic death through cyclin B1 accumulation in the nucleus.^23^ We therefore reasoned that inappropriate cyclin B1 upregulation may be involved in isoflurane‐induced neuronal degeneration.

The cyclin B1‐Cdk1 complex in the mitochondria acts upstream of antiapoptotic protein Bcl‐xL.^23^ The Bcl‐xL is a member of the Bcl‐2 family, which plays a critical role in promoting cell survival by regulating mitochondrial membrane permeabilization.^29^ Conditional deletion of Bcl‐xL in the brain induces apoptosis in the upper layer cortical neurons at the early postnatal stages.^32^ Previous study found that isoflurane exposure for 6 hours increases the expression of pro‐apoptotic protein Bax, decreases the expression of antiapoptotic protein Bcl‐2, facilitates release of cytochrome *c* from the mitochondria, and induces apoptosis in adult mouse.^33^ The effect of isoflurane on brain Bcl‐xL expression in postnatal rodent has not been investigated so far. Our data show increased levels of p‐Bcl‐xL and cytochrome C at 6 hours after discontinuation of isoflurane. Administration of CR8 significantly reduced the levels of the p‐Bcl‐xL, cytochrome C, and cleaved caspase‐3 in a manner that was correlated with the inhibition of cell cycle activation, suggesting a link between cell cycle arrest and neuronal cell death in the brain after isoflurane.

Over the past years, several groups have explored the possible role of cell cycle in general anesthetics induced neurodegeneration in the development brain. Anesthetic‐induced upregulation of neuronal cell cycle proteins was firstly reported in 7‐day‐old rats with an N‐Methyl‐d‐Aspartate (NMDA) receptor antagonist ketamine.^34^ Repeated administration of ketamine over 6 hours increases expression of cell cycle protein cyclin D1, cdk‐4, E2F1, and Bim, which in turn provokes apoptosis through the intrinsic pathway. Administration of small interfering RNA (siRNA) targeting cyclin D1 inhibits cell apoptosis from ketamine exposure in vitro.^34^ Current evidence of isoflurane exposure on cell cycle signaling was not consistent. In one study, 1.5% isoflurane treatment for 4 hours was found to result in an aberrant CDK5 activation and neuronal apoptosis in both rat pups in vivo and hippocampal neuronal cultures in vitro. Inhibition of CDK5 attenuates neuronal cell death and learning/ memory impairments.^35^ In another study, 7‐day‐old mice with 0.75% isoflurane for 6 hours induces significantly increased apoptosis cell death without significant change in cell cycle regulatory proteins (CDK4, cyclin D1).^5^ These findings suggest that isoflurane may require threshold concentration to induce neuronal cell cycle activation and that aberrant cell cycle reentry is another pathway rather than the primary mediator of isoflurane‐induced neuronal apoptosis.^34^

The current study evaluated the neuroprotective efficacy of a pharmacological cyclin‐dependent kinases (CDKs) inhibitor CR8. CR8 is a N6‐biaryl‐substituted derivative of roscovitine, which has been shown to remarkably alleviate neurodegeneration, learning and memory impairment induced by postnatal isoflurane exposure.^35^ However, the therapeutic potential of roscovitine is confined by rapid metabolic deactivation and a short biological half‐life.^36^ In addition, its potency for inhibition of purified CDKs and CDK activity in cell lines is relatively weak. CR8 has enhanced inhibition of purified CDK1/2/3/5/7/9, improved solubility, cell permeability, and intracellular stability, leading to about 68‐fold more potency than roscovitine in various cell lines in vitro.^36^ Studies have demonstrated that CR8 significantly attenuate neuronal cell cycle activation and progressive neurodegeneration in multiple models of experimental traumatic brain injury (TBI) and spinal cord injury.^19^, ^36^, ^37^, ^38^

In conclusion, our results identify a cascade of events triggered by isoflurane exposure in the developing brain. This transduction pathway includes upregulation of neuronal cell cycle protein cyclin B1, activation of Cdk1, and phosphorylation of antiapoptotic protein Bcl‐xL. Phosphorylated Bcl‐xL initiates cytochrome c release and caspase‐3 activation that results in apoptotic cell death. Furthermore, we demonstrated for the first time that the selective CDKs inhibitor CR8 confers protection against isoflurane‐induced cell cycle activation and neurodegeneration. These findings suggest that use of cell cycle inhibitors may provide a possible therapeutic target for prevention of developmental anesthetic neurotoxicity.

## CONFLICT OF INTEREST

The authors declare that there is no conflict of interest.
